# Relevance of Plasma Homocysteine and Methylenetetrahydrofolate Reductase 677TT Genotype in Sickle Cell Disease: A Systematic Review and Meta-Analysis

**DOI:** 10.3390/ijms232314641

**Published:** 2022-11-24

**Authors:** Paul R. J. Ames, Alessia Arcaro, Matilde Caruso, Maria Graf, Vincenzo Marottoli, Fabrizio Gentile

**Affiliations:** 1 Immune Response and Vascular Disease Unit, Centro de Estudos de Doenças Crónicas, Universidade Nova de Lisboa, 1150-082 Lisbon, Portugal; 2 Department of Haematology, Dumfries Royal Infirmary, Dumfries DG2 8RX, UK; 3 Department of Medicine & Health Sciences ‘V. Tiberio’, Università del Molise, 86100 Campobasso, Italy; 4 Transfusion Medicine Unit, Cardarelli Hospital, 86100 Campobasso, Italy; 5 Immunohaematology and Transfusion Medicine Unit, Department of Laboratory and Transfusion Medicine, Federico II University Hospital, 80131 Napoli, Italy; 6 Multimedica Srl, 80134 Naples, Italy

**Keywords:** homocysteine, methylenetetrahydrofolate reductase, MTHFR TT genotype, sickle cell disease, vaso-occlusive crisis, ischemic stroke

## Abstract

We evaluated the relevance of plasma homocysteine (HC) and the TT genotype of the methylenetetrahydrofolate reductase (MTHFR) C677T polymorphism (rs1801133) in sickle cell disease (SCD) and associated vaso-occlusive crisis (VOC) and ischemic stroke (IS). We identified in Embase and Medline 22 studies on plasma HC and 22 on MTHFR genotypes. Due to age-related HC differences, adult and paediatric SCD were separated: 879 adult SCD and 834 controls (CTR) yielded a neutral effect size; 427 paediatric SCD and 625 CTR favoured SCD (*p* = 0.001) with wide heterogeneity (*I*^2^ = 95.5%) and were sub-grouped by country: six studies (Dutch Antilles *n* = 1, USA *n* = 5) yielded a neutral effect size, four (India *n* = 1, Arab countries *n* = 3) favoured SCD (*p* < 0.0001). Moreover, 249 SCD in VOC and 419 out of VOC yielded a neutral effect size. The pooled prevalence of the MTHFR TT genotype in 267 SCD equalled that of 1199 CTR (4.26% vs. 2.86%, *p* = 0.45), and in 84 SCD with IS equalled that of 86 without IS (5.9% vs. 3.7%, *p* = 0.47); removal of one paediatric study yielded a significant effect size (*p* = 0.006). Plasma HC in paediatric SCD from Middle East and India was higher, possibly due to vitamin deficiencies. Despite its low prevalence in SCD, the MTHFR TT genotype relates to adult IS.

## 1. Introduction

Sickle cell disease (SCD) is a severe haemoglobinopathy characterised at the molecular level by a valine to glutamic acid substitution at position 6 in the β-globin chain: the homozygous mutation affects both β-globin chains yielding haemoglobin (Hb) SS that at low oxygen tension polymerises into a fibrous mesh that changes the normal discoid shape of red cells into a sickle shape [1]. At the clinical level, the erythrocyte shape change leads to acute vaso-occlusive crises (VOC) in the microcirculation that are typical of SCD [2]; as more crises accrue, patients may develop a chronic vasculopathy within the pulmonary, the cerebral, and the peripheral circulation, leading to pulmonary hypertension, ischaemic strokes (IS), and leg ulcerations [3]. Moreover, SCD patients have a greater risk of venous thromboembolism than the general population [4]. Additional factors that contribute to the VOC are the intravascular haemolysis and the neutrophil–platelet aggregates in the pulmonary circulation, both of which cause oxidation [5,6], complement, and coagulation activation [7,8].

Homocysteine (HC) is a sulphur-containing amino acid whose fate is either re-methylation to methionine or trans-sulphuration to cystathionine according to the different enzymes that control the two pathways: in particular, a polymorphism in the methylenetetrahydrofolate reductase (MTHFR) C677T gene (rs1801133) codes for an enzyme that has reduced activity to convert 5,10-methylenetetrahydrofolate to 5-methyltetrahydrofolate generating less methyl groups required for the re-methylation of HC to methionine; therefore, HC reaches toxic intracellular and plasma concentrations ultimately favouring thrombosis [9] and vascular damage [10]. The present systematic review and meta-analysis explores the possible contribution of plasma HC and of the MTHFR genotypes to SCD and some of its clinical manifestations.

## 2. Results

**Figure 1:**
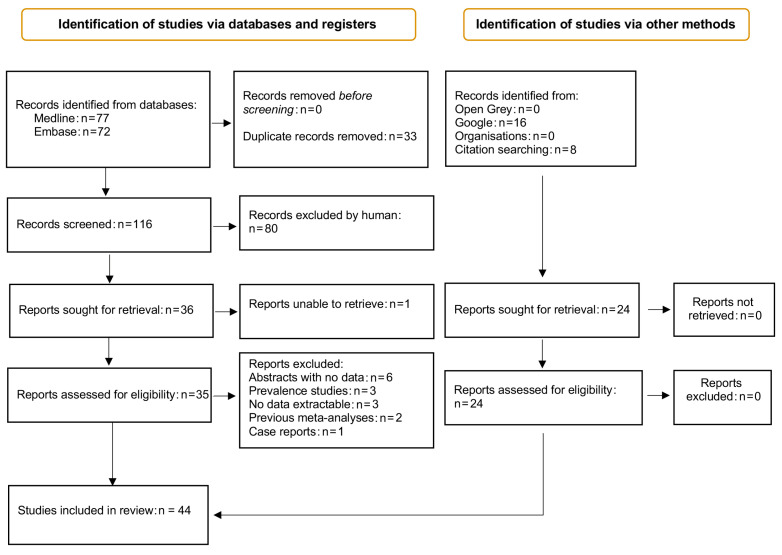
Prism flowchart indicating the screening and exclusion process of articles up to final inclusion in the qualitative and quantitative analysis.

The database search identified 149 citations; as shown in our flowchart (Figure 1), we finally considered 44 articles, 42 of which were full papers [11,12,13,14,15,16,17,18,19,20,21,22,23,24,25,26,27,28,29,30,31,32,33,34,35,36,37,38,39,40,41,42,43,44,45,46,47,48,49,50,51,52] plus one thesis [53] and one abstract [54] that examined the relationship between HC, MTHFR, and SCD and that were included in our systematic review and meta-analysis. In particular, Table 1 shows the studies that investigated plasma HC in SCD (22 case-control and 2 cohort studies), Table 2 shows the studies that investigated MTHFR TT and cystathionine beta synthase (all case-control), and Table 3 shows the studies that investigated the MTHFR TT genotype in relation to clinical features of SCD (all cohort studies).

### 2.1. Effect Size of Homocysteine in Sickle Cell Disease

Pooled data from 22 case control articles yielded 1269 SCD participants and 1481 controls; the effect size favoured SCD (*p* = 0.009) with wide heterogeneity (*I*^2^ = 96.2%, *p* < 0.0001) (Supplementary Figure S2): sensitivity analysis using age as a moderator factor indicated that the effect size (the average standard difference between HC means) favoured paediatric rather than adult participants (coefficient −0.068, 95% CI −0.117, −0.019, *p* = 0.006). We therefore examined the two groups separately.

### 2.2. Effect Size of Homocysteine in Adult Sickle Cell Disease

**Figure 2:**
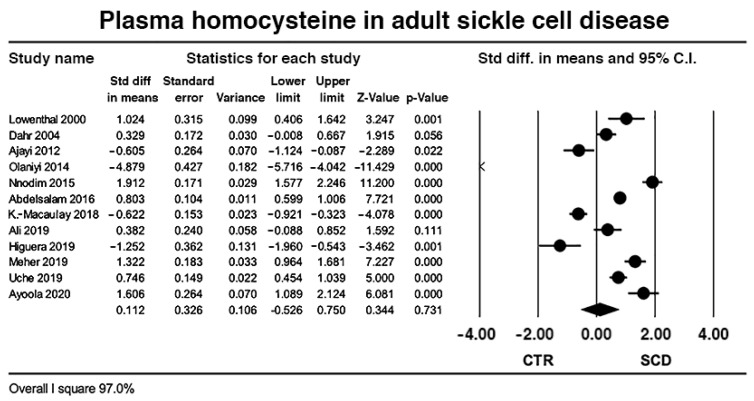
Effect size of studies comparing plasma homocysteine in control and adult sickle cell disease.

Pooled data from 12 case control studies yielded 879 adult SCD and 834 controls; the effect size was neutral with wide heterogeneity (*I*^2^ = 95.6%, *p* < 0.0001) (Figure 2). Sensitivity analysis by meta-regression including year of publication, sample size, mean age of SCD participants, female to male ratio, and NOQAS, changed neither effect size nor heterogeneity (Table 4A). Sensitivity analysis by subgroups including ethnicity of the SCD patients, methods of HC determination, B12 and folic acid measurement, presence, and absence of VOC, revealed a slightly decreased heterogeneity by method of HC measurement, but no change in effect size (Table 4B).

### 2.3. Effect Size of Homocysteine in Childhood Sickle Cell Disease 

**Figure 3:**
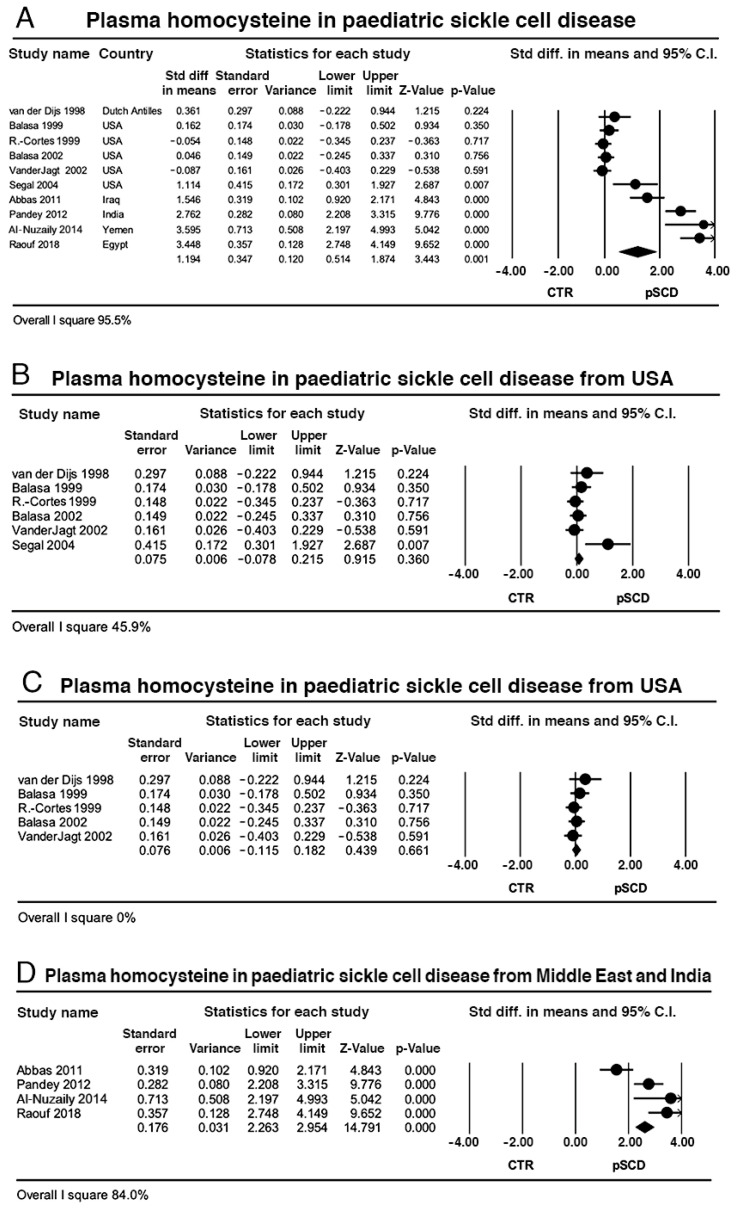
(**A**) Effect size of studies comparing plasma homocysteine in control and in paediatric sickle cell disease; (**B**) subgroup analysis on one study from the Dutch Antilles and the five studies from USA; (**C**) subgroup analysis as in B after removal of the outlier study from USA; (**D**) subgroup analysis on three studies from Arab countries and one from India.

Pooled data from 10 case-control studies yielded 427 children with SCD and 625 controls: the effect size favoured SCD (*p* = 0.001) with wide heterogeneity (*I*^2^ = 95.5%, *p* < 0.0001) (Figure 3A). Sensitivity analysis by meta-regression showed that the sample size, female to male ratio and mean age of participants slightly explained the heterogeneity variance (Table 5A) as well as the methods of HC determination (Table 5B).

Subgroup analysis on the study from the Dutch Antilles [11] and the five studies from USA [12,13,15,16,18] revealed a neutral effect size with moderate heterogeneity (*I*^2^ = 45.9%, *p* = 0.1) (Figure 3B) that fully disappeared after removal of the one outlier study that favoured the effect size [18] (Figure 3C). Instead, subgroup analysis on the three studies from Arab countries [19,22,27] and one from India [21] revealed a significant effect size with elevated heterogeneity (*I*^2^ = 84%, *p* < 0.0001) (Figure 3D). Of these four studies, one reported normal average serum B12 and folate concentrations [19], one reported a low average folate but did not measure B12 [27], and the other two measured neither vitamin [21,22]; when we grouped together the latter two studies, the heterogeneity dropped to low (*I*^2^ = 15.2%, *p* = 0.15) with a significant effect size (*p* < 0.0001), though the studies were carried out in relatively distant countries, India [21] and Yemen [22], respectively.

### 2.4. Effect Size of Homocysteine on Vaso-Occlusive Crisis 

We pooled data from two paediatric [12,33] and six adult studies [23,24,26,29,32,53] comprising 249 participants in crisis and 419 unmatched participants in steady state; two studies included patients with IS as part of their VOC, one dealing with children [12] and one with a mix of children and adults [32], both from USA. The effect size was neutral with wide heterogeneity (*I*^2^ = 91.7%, *p* < 0.0001) (Supplementary Figure S3A). After removal of the study from India [29], the effect size remained neutral with a slightly reduced heterogeneity (*I*^2^ = 87.1%, *p* < 0.0001) (Supplementary Figure S3B); further removal of the studies from Nigeria [23,24,53] shifted effect size to steady state SCD with reduced heterogeneity (*I*^2^ = 52.7%, *p* = 0.09) (Supplementary Figure S3C).

### 2.5. Effect Size of Methylenetetrahydrofolate Reductase in Sickle Cell Disease 

**Figure 4:**
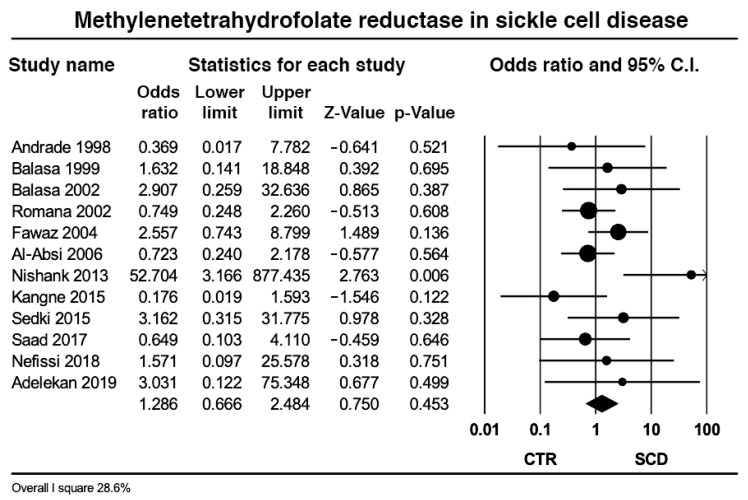
Effect size of the pooled prevalence of the methylenetetrahydrofolate reductase TT in controls and in sickle cell disease.

Data from 12 studies including 1267 SCD patients and 1199 controls revealed that the pooled prevalence of the MTHFR TT genotype was relatively similar in the two groups (4.26% vs. 2.86%, *p* = 0.45) with low heterogeneity (*I*^2^ = 28.6%, *p* = 0.16) (Figure 4); subgroup analysis on the three studies from the Americas [12,15,35] yielded nil heterogeneity (*I*^2^ = 0%, *p* = 0.54), with a neutral effect size (plot not shown); likewise, subgroup analysis on the four African studies [40,41,42,54] yielded nil heterogeneity (*I*^2^ = 0%, *p* = 0.89) and neutral effect size; subgroup analysis on the two Arab studies [36,37] and on the two Indian studies [38,39] yielded medium (*I*^2^ = 56.8%, *p* = 0.12) and high heterogeneity (*I*^2^ = 90.5%, *p* = 0.001), respectively, without changing the effect size.

Data from three studies including 237 SCD patients and 351 controls revealed that the pooled prevalence of the MTHFR 1298CC genotype (rs10948059) in SCD was double of that of controls (9.7% vs. 4.2%, *p* = 0.047) with low heterogeneity (*I*^2^ = 12.4%, *p* = 0.31) (Supplementary Figure S4).

### 2.6. Effect Size of Cystathionine Beta Synthase in Sickle Cell Disease 

Data from two studies including 370 SCD patients and 245 controls revealed a similar pooled prevalence of the homozygous CBS in68 between the two groups (1.9% vs. 1.2%, *p* = 0.83), with medium heterogeneity (*I*^2^ = 28.7%, *p* = 0.23).

### 2.7. Effect Size of Methylenetetrahydrofolate Reductase in Vaso-Occlusive Crisis 

**Figure 5:**
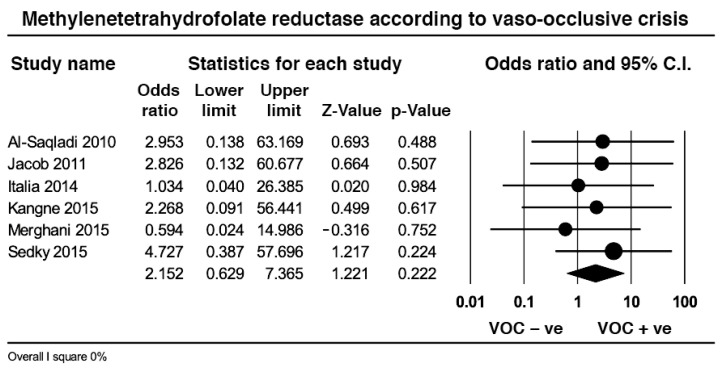
Effect size of the pooled prevalence of the methylenetetrahydrofolate reductase TT in sickle cell disease out and in vaso-occlusive crisis.

Data from six studies including 321 patients in crisis and 228 out of crisis revealed that the pooled prevalence of the MTHFR TT genotype was slightly higher in VOC than in the steady state (2.41% vs. 0.87%, *p* = 0.22) with no heterogeneity (Figure 5).

### 2.8. Effect Size of Methylenetetrahydrofolate Reductase in Ischaemic Stroke 

**Figure 6:**
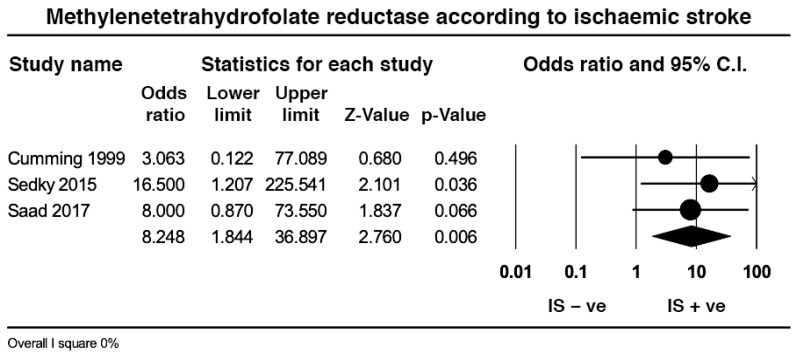
Effect size of the pooled prevalence of the methylenetetrahydrofolate reductase TT in sickle cell disease without and with ischemic stroke.

Six studies investigated the relation between ischemic stroke (IS) and MTHFR TT: two of these had no MTHFR TT genotypes in the positive and negative IS groups [34,44] and were not considered. Hence, data from four studies including 84 SCD patients with ischemic stroke and 186 without ischemic stroke revealed a similar pooled prevalence of MTHFR TT between the two groups (5.9% vs. 3.7%, *p* = 0.47) with medium heterogeneity (*I*^2^ = 32.6%, *p* = 0.21); however, removal of the study with the youngest participants [49] revealed a significant effect size (*p* = 0.006) with no heterogeneity (Figure 6). Two studies did not report the age of the participants [40,54].

### 2.9. Effect Size of Methylenetetrahydrofolate Reductase in Avascular Necrosis 

Data from two studies including 52 patients with avascular necrosis of the femoral heads and 76 patients without such feature a higher pooled prevalence of the MTHFR TT genotype in the latter group (3.9% vs. 1.97%, *p* = 0.66) with no heterogeneity (*I*^2^ = 0) [plot not shown].

### 2.10. Age at Presentation of Vaso-Occlusive Crisis

Three studies revealed an earlier age at VOC presentation in MTHFR TT carriers: the first showed that 78.1% (50/64) of MTHFR TT patients developed the 1st VOC between 0–3 years of age, compared to only 6.9% (6/86) of wild types within the same age range [38]; the second revealed a median age at onset of any VOC at 15 months in MTHFR TT (*n* = 2) compared to 42 months in wild type (*n* = 12) [40] and the third revealed a median age at onset of any VOC at 6 months of age in MTHFR TT (*n* = 3) compared to 24 months of age in wild type (*n* = 19) [54]. The data of these three studies could not be pooled because of their incomplete data representation and different data expression; in particular, on the website of the relevant journal all pages were split into two halves, one half-printed, one half-blank [40].

## 3. Discussion

Our preliminary sensitivity analysis revealed a different behaviour between adult and paediatric SCD; hence, we carried out the meta-analysis on these two populations separately. With regards to adult SCD, the overall effect size was neutral with a high heterogeneity unexplained by the extensive sensitivity analysis, but for a slight effect of the methods of plasma HC measurements; this consistency favours the reliability of the SCD/control comparison as the heterogeneity remained elevated for each of the explanatory factors evaluated, but it leaves unresolved the issue of HC in adult SCD.

The method of HC determination marginally explained the heterogeneity of paediatric SCD, but the subgroup analysis revealed that the standardized mean difference in plasma HC between patients and controls from USA was neutral, supported by the lack of heterogeneity between studies; at variance, the three studies from Arab countries [19,22,27] and the one from India [21] revealed a significant effect size with elevated heterogeneity. An effect of vitamin B deficiency cannot be ruled out as one study declared normal average serum B12 and folate serum concentrations [19] and one reported a low average folate without measuring vitamin B12 [27] while the remaining two measured neither vitamin [21,22].

Although the intracellular and plasma HC concentrations are genetically determined, they are also influenced by environmental factors, such as age, gender, lifestyle, nutrition, physical activity, smoking, and medication [55]. In this respect, food fortification with folic in USA could explain the lack of heterogeneity in the paediatric studies from USA though the fortification occurs unevenly across ethnic groups, being less valid in Afro Caribbeans [56], and it does not necessarily translate into lower plasma HC concentrations [57], as food insecurity is still an issue for the SCD population in the USA [58]. On the other hand, a decade-old meta-analysis revealed that malnutrition and under-nutrition are common in SCD children from the Middle East, possibly a consequence of the poor knowledge of the nutritional status of Arab paediatric patients [59]; this reflects in micro and macro nutritional deficiencies leading to greater disease severity and poorer quality of life, a situation not dissimilar from adult Arab SCD patients [60]. A previous meta-analysis on the relation between HC and SCD concluded that plasma HC could be considered a bio-marker of SCD as the calculated effect size favoured SCD, but the authors investigated neither the source of the heterogeneity nor performed any subgroup analysis, leaving their results unsupported and open to criticism [61].

With regards to VOC, our meta-analysis found a neutral effect size between SCD children and controls, the wide heterogeneity decreasing after removal of the studies from India [29] and from Nigeria [23,24,53], implying that these latter contributed to the heterogeneity, possibly via the same nutritional and vitamin deficiencies alluded to earlier [62]. It should be noted that in the VOC comparison, SCD patients were unmatched, preventing the capture of the same genetic, oxidative, and nutritional background of the participants before and after crisis, that might have allowed a better interpretation of the results.

With regards to MTHFR TT, the pooled prevalence of this genotype was low and relatively similar between SCD and controls, and even if the prevalence of the other MTHFR 1298CC genotype was double of that of controls, the contribution of both genes to the clinical manifestations of SCD remains dubious; having excluded a study that mixed adult and paediatric patients [49], the pooled prevalence of MTHFR TT in patients with ischemic stroke was higher than non-stroke controls, but still at a relatively low 5.9%. The pooled prevalence of MTHFR TT in patients with any VOC was 2.4%, non-significantly higher than patients in steady state whereas the pooled prevalence of MTHFR TT was lower in patients with avascular necrosis than in patients without.

In a two-year-old meta-analysis on the same topic [63], the author used the recessive allelic frequency [64] to demonstrate a significant 1.81 odds ratio of developing any VOC with low heterogeneity. Our effect size for any VOC was neutral and without heterogeneity, but the effect size for ischemic stroke was significant and devoid of heterogeneity. Given that three of the articles on ischemic stroke were present in the previous meta-analysis [40,45,49], one wonders whether a subgroup analysis would have modified the author’s conclusions [64].

Overall, our meta-analysis supports neither an involvement of plasma HC in SCD and its clinical manifestations, nor a definite role for the MTHFR TT genotype, the pooled prevalence of which is low, even if associated with ischemic stroke.

However, this does not mean that MTHFR has no relevance in SCD: a recent article demonstrated that the co-inheritance of HbSS and MTHFR TT negatively affects the antioxidant capacity of SCD patients [65]: indeed, a low MTHFR activity reduces the production of 5-methyl tetrahydrofolate, leading to lower plasma and erythrocyte folate concentrations [66] and to a decreased antioxidant effect against superoxide anion [67].

It has been noted that when the MTHFR TT genotype, itself associated with oxidative stress, is present in patients with other diseases characterised by oxidative stress, it may contribute to an earlier age at onset of the other disease. This is the case of the primary antiphospholipid syndrome: two separate cohorts from Southern Italy show that primary antiphospholipid antibody (PAPS) patients carrying the MTHFR TT genotype developed their vascular occlusion 16 years and 27 years earlier than PAPS carriers of MTHFR CT + CC [68,69]. Similarly, MTHFR TT positive patients suffering from multiple sclerosis, a disease characterised by oxidation [70], developed their disease below 30 years of age, 4 years earlier than MTHFR CC+CT patients [71].

While scrutinising the citations for this systematic review, we came across this anticipation phenomenon in three articles [38,39,40]. Therefore, despite a low prevalence of MTHFR TT in SCD, this genotype may still affect morbidity and quality of life at a significantly earlier age. This anticipation phenomenon deserves an interpretation.

A purely vascular interpretation may derive from studies showing that cultured human venous endothelial cells (HUVEC) exposed to high concentrations of extracellular HC generate HC-thiolactone, an active metabolite able to acylate free amino groups allowing the incorporation of HC into proteins in a process called homocysteinylation [72]; such post-translationally modified proteins lose their functions and acquire cytotoxic and pro-inflammatory properties, contributing to the atherothrombotic tendency associated with severe hyperhomocysteinemia [73].

A purely erythrocytic interpretation may derive from the observation that intra-erythrocyte Hb S recycling between ferric and ferryl iron generates an oxidative environment conducive to irreversible post-translational modification of the βCys93 into cysteic acid and to the ubiquitination of the Hb β-Lys-96 and β-Lys-145 side chains and of the mitochondria [74,75]; whether elevated intra-erythrocytic HC induces S-homocysteinylation of βCys93 and contributes to premature sickling is an attractive hypothesis not tested so far.

Moreover, intracellular HC may induce endoplasmic reticulum stress, that up-regulates MTHFR via the transcriptional activator NF-κB, but if the MTHFR TT is up-regulated, the endoplasmic stress will not be quenched by a reduction of intracellular HC, rather it will be sustained and further contribute to a decreased intracellular antioxidant capacity [76].

Additionally, the coincidental oxidative [77] and nitrative stress [78] that characterise SCD, particularly during active crisis, can inhibit cystathionine beta synthase [79], preventing the entry of HC through the trans-sulphuration pathway; this will lead to elevation of intracellular and plasma HC that at toxic concentrations may further inhibit CBS via a disulphide redox mechanism [80], perpetuating its own elevation and eventually contributing to recurrent VOC and to long term vascular damage [3,7], the latter characterized amongst others by ischemic stroke even in the paediatric age range [81].

Our meta-analysis has several limitations: (1) many studies included a mix of HbSS, HbSC, and HbS-β_0_thal that may have weakened certain relationships; (2) plasma HC was measured only once in all articles, precluding the assessment of its persistence and therefore of its long-term clinical consequences; (3) the studies on VOC compared unmatched patients in and out of crisis, weakening the value of the comparison; (4) plasma HC and the MTHFR genotypes have not been evaluated with regards to SCD vasculopathy; (5) we cannot discount a degree of publication bias, the evaluation of which by an empirical graphical method can be misleading and inappropriate for observational studies [82,83].

## 4. Methods

### 4.1. Search Strategy

For the purpose of the systematic review, the Medline database was screened from inception to July 2022 using the Medical Subject Headings (“sickle cell disease”[All fields] OR “sickle cell anemia”[All fields] AND (“homocysteine”)[All fields] AND (“methylenetetrahydrofolate reductase” [All fields]) AND (“cystathionine beta synthase”) [All fields]; the EMBASE database was screened from inception to present with “sickle cell disease” OR “sickle cell anemia”/exp AND “homocysteine”/exp AND “methylenetetrahydrofolate reductase”/exp AND “cystathionine beta synthase”/exp. To reduce the effect of possible publication bias, we used the same search terms in natural language to screen the Grey Literature via the DANS EASY Data Archive, as well as Google, looking for additional citations. We finally hand-searched the reference list of all papers subsequently included in the systematic review to ensure we had not missed any relevant articles.

### 4.2. Inclusion Criteria

We included in our meta-analysis: (1) retrospective, cross-sectional, and prospective case-control or cohort studies addressing the difference in mean plasma HC between SCD patients and controls (CTR) or between patients with and without different clinical manifestations of SCD, as well as the prevalence of MTHFR and CBS polymorphisms between SCD and CTR or between patients with and without different clinical manifestations of SCD; (2) studies in which plasma HC was measured by validated and published method; (3) articles written in any language.

### 4.3. Exclusion Criteria

We excluded from our meta-analysis: (1) case studies, prevalence studies, and reviews; (2) articles not comparing SCD patients with healthy CTR; (3) plasma HC not measured with validated methods. Two investigators (PRJA and AA) checked independently the resulting citations for relevancy and removed duplicates (via EndNote); A.A., M.C., M.G., and V.M. screened all titles and abstracts, excluded the irrelevant ones, and applied the eligibility criteria to the relevant ones in order to include the appropriate studies. P.R.J.A. and F.G. also screened the reference list of retrieved papers for papers that could have been missed.

### 4.4. Data Extraction

A.A., M.C., M.G., and V.M. independently extracted data from the articles that considered: year of publication, study design, sample size, demographic data, SCD subtype, follow-up, outcome means, and corresponding dispersion measures (standard deviations or confidence intervals). The 2020 PRISMA guideline was followed to ensure transparency of identification, selection, appraisal, and synthesis of the studies included in the systematic review and meta-analysis [84]. We did not subscribe the systematic review to a registry because our data derive from case-control observational studies with no intervention, with no specified protocol other than what was extracted as described Table 1, Table 2 and Table 3.

### 4.5. Evaluation of the Quality of the Studies

The quality of the studies included in the meta-analysis was assessed by the Newcastle Ottawa Quality Assessment Scale (NOQAS) for observational case-control and cohort studies [85]. The three major domains (selection of cases and controls, comparability of the groups and verification of either the exposure or outcome of interest) yield a score ranging between 0 and 8, the higher the score the better the methodological quality. F.G. and VM independently scored the selected articles and input the results into an electronic form; any discrepancies were resolved by consensus or via a third party (P.R.J.A.). The inter-rater agreement between the two assessors was high (Cohen kappa 0.74, 95% CI 0.697, 0.880).

### 4.6. Outcome Measures

The primary outcomes were the pooled standardized mean differences of HC between SCD patients and healthy controls and within different clinical subgroups of SCD; the secondary outcome was the difference in the pooled prevalence of subjects with different MTHFR polymorphisms between SCD and healthy controls or between SCD with different clinical manifestations.

### 4.7. Statistical Analysis

The statistics was carried with the Comprehensive Meta-analysis software (Version 3, Englewood, NJ 2013, USA). Since the estimates derived from observational studies, we employed random effects meta-analyses for continuous outcomes [86] and Peto’s odds ratio to compare prevalence between groups as it performs well with rare events [82]. Heterogeneity between study results was evaluated by the *I*^2^ statistics: an *I*^2^ value of 0% indicates no heterogeneity; values less than 25% indicate low, between 25% and 50% moderate, and over 50% high heterogeneity [83]. Sensitivity analyses were not predefined at this stage but investigated according to heterogeneity. Publication bias was assessed by the empirical funnel plot (Supplementary Figure S1) [87,88].

## 5. Conclusions

The minimal heterogeneity of plasma HC in children from USA compared to children from the Middle East and India suggest that geographical factors linked to local nutritional patterns may account for this difference, though individual studies were insufficiently powered to address the number of factors that may influence plasma HC [56]. The MTHFR TT genotype seems related to IS, but whether the latter developed in the context of pre-existing cerebral vasculopathy or as an ex-novo occurrence in unaffected cerebral vessels is unclear. Despite the low prevalence of MTHFR TT, the latter genotype might affect age at onset of VOC. All these issues should be re-evaluated on properly designed prospective studies that should take into account the knowledge on the regulation of the enzymatic pathways that control intracellular HC levels.

## Figures and Tables

**Table 1:** Demographics and clinical features of the case control and cohort studies on plasma homocysteine.

Ref.	Author/Year	Ethnicity	CTR	F/M	Age	HC	SCD		F/M	Age	HC	HC	FA	B12	VOC	IS	HU	NOS
							SS	SC/βthal										
			No.	No.	Years	μmol/L	No.	No.	No.	Years	μmol/L	Method						
					*x* ± SD	*x* ± SD				*x* ± SD	*x* ± SD							
**Case control studies**																		
[11]	van der Dijs 1998	Dutch Antilles	20	10/10	9 ± 4	10.9 ± 3.5	27		15/12	8 ± 4	12.5 ± 5	HPLC	nor	nor				7
[12]	Balasa 1999	USA	198		8.3 ± 4.9	6 ± 3.1	40			12.8 ± 6.2	6.5 ± 3	HPLC	nor	nor	y	y		6
[13]	Rodriguez-Cortes 1999	USA	73			6.1 ± 2.7	120		51/69	10.5 ± 5	5.2 ± 2.5	HPLC	nor	na	y			6
[14]	Lowenthal 2000	USA	16		36 ± 12	9.7 ± 3.7	37	12	30/19	30.5 ± 9	16.8 ± 7.9	HPLC	high	nor				5
[15]	Balasa 2002	USA	110	63/47	10.8 ± 4.29	7.5 ± 2.1	77		43/34	11.1 ± 5.2	8.25 ± 3.4	HPLC	nor	B6		y		5
[16]	VanderJagt 2002	USA	77	40/37	13.3 ± 3.0	9.9 ± 5.6	77		37/40	13.4 ± 3.6	9.5 ± 3.35	FPIA	na	na				4
[17]	Dhar 2004	USA	75	50/25	42 ± 13	8.5 ± 3.1	63	8/9	63/27	37 ± 12	9.7 ± 4.2	ELISA	high	nor			y	7
[18]	Segal 2004	USA	11	8/3	8.3 ± 3.7	4.3 ± 1.03	17		9/8	9.8 ± 3.9	5.4 ± 0.96	HPLC	nor	nor			exc	6
[19]	Abbas 2011	Iraq	25	13/12	14.4 ± 7.69	18.65 ± 4.56	6	0/20	10/16	14.3 ± 7.6	44.52 ± 23	HPLC	nor	nor				5
[20]	Ajayi 2012	USA	57	33/24	464 ± 14	9.12 ± 0.9	20	7/2	9/20	34 ± 10	8.35 ± 2	na	high	na			y	8
[21]	Pandey 2012	India	60	23/37	11.2 ± 5.3	8.7 ± 4.25	40		16/24	11.2 ± 5.3	25.7 ± 8.24	ELISA	na	na				5
[22]	Al-Nuzaily 2014	Yemen	20	8/12	8.6 ± 4.6	8.9 ± 1.8	5		1/4	6.9 ± 3.0	20.8 ± 6.9	ELISA	na	na				5
[23]	Olaniyi 2014	Nigeria	30	16/14	26 ± 4.8	9.13 ± 0.75	60		28/32	26 ± 5	5.79 ± 0.65	HPLC	low	low	y			6
[24]	Nnodim 2015	Nigeria	100			13.6 ± 4.8	100			5–30	24.2 ± 6.2	Spectro	na	na	y			4
[25]	Abdelsalam 2016	Sudan	200		26.25 ± 5.25	4.92 ± 1.77	200			29.5 ± 5.5	6.47 ± 2.08	ELISA	na	na				4
[26]	Knox-Macaulay 2018	Oman	151	27/114	26.6 ± 10.7	11.55 ± 5.9	32	0/101	73/60	21 ± 5.7	8.05 ± 2.4	FPIA	nor	nor	y		exc	6
[27]	Raouf 2017	Egypt	30	13/17	6.03 ± 2.64	18.8 ± 3.7	18	0/32	17/33	6.2 ± 2.5	44.6 ± 9	HPLC	low	nor	y			5
[53]	Ali 2019	Nigeria	26	12/14	27.6 ± 6.6	9.9 ± 2.5	55		28/27	24.8 ± 5.5	11.1 ± 3.4	ELISA	nor	nor	y			6
[28]	Higuera 2019	Venezuela	23	13/10	33 ± 7	9.43 ± 1.8	15		10/5	31 ± 7.6	6.97 ± 2.2	FPIA	na	na				5
[29]	Meher 2019	India	50	32/18	21 ± 4	13.2 ± 4.4	120		61/59	24 ± 8	22.41 ± 7.78	Spectro	na	na	y		exc	7
[30]	Uche 2019	Nigeria	96	51/45	30 ± 11	9.16 ± 4.29	96		51/45	29 ± 12	19.8 ± 19.7	ELISA	na	na	y			5
[31]	Ayoola 2020	Nigeria	33	16/17	24 ± 3	10.2 ± 4.1	44	3/0	21/23	25 ± 3.7	17.95 ± 5.3	ELISA	na	na				6
**Cohort studies**																		
[32]	Houston 1997	USA					99		53/46	19	11.1 ± 4	HPLC	nor	na		y		6
[33]	Al-Saqladi 2010	Yemen					102		46/56	7.2	2.8 ± 1.7	EIA	nor	nor				5

Abbreviations. Ref.: reference; No.: number; CTR: controls; F/M: female/male; HC: homocysteine; SCD: sickle cell; FA: folic acid; VOC: vaso-occlusive crisis; IS: ischaemic stroke; HU: hydroxyurea; NOS: Newcastle–Ottawa Scale; SD: standard deviation; SS: homozygous haemoglobin S; SC/βthal: hemoglobin SC/βthalassaemia; na: not available; nor: normal; exc: excluded; HPLC: high-performance liquid chromatography; FPIA: fluorescence polarisation immunoassay; ELISA: enzyme-linked immunosorbent assay; Spectro: spectrometry; EIA: enzyme immunoassay.

**Table 2:** Demographics and clinical features of the case control studies on methylenetetrahydrofolate reductase TT and cystathionine beta synthase genotypes.

Ref.	Author/Year	Ethnicity	CTR	F/M	Age	M-TT	SCD		F/M	Age	M-TT	VOC	NOS
							SS	SC/βthal					
			No.	No.	Years	No.	No.	No.	No.	Years	No.		
					*x* ± SD					*x* ± SD			
[34]	Andrade 1998	Brazil	137			2	73	53/20	40/33	35 ± 13	0		4
[35]	Romana 2002	Guadalupe	203			6	314	314			7	y	5
[36]	Fawaz 2004	Saudi Arabia	105	40/65	32.2 ± 15	4	87	87	49/38	23.1 ± 14.1	8		5
[37]	Al-Absi 2006	Bahrain	156	76/80	27.8 ± 15.1	10	106	106	38/68	15.8 ± 9.8	5		4
[38]	Nishank 2013	India	150		17 ± 6.8	0	150	150		16 ± 6	22		5
[39]	Kangne 2013	India	130		16.5 ± 11	4	180	126/54	78/102	16.5 ± 11	1	y	5
[40]	Sedki 2015	Egypt	40			1	40				3	y	4
[54]	Saad 2017	Egypt	40			3	40	13/27			2	y	NE
[41]	Nefissi 2018	Tunisia	100			1	64	35/29	38/26	3–27	1		6
[42]	Adelekan 2019	Nigeria	96	51/45	29.3 ± 10.3	0	96	96	51/45	29.3 ± 10.3	1		7
						**CBSin68**					**CBSin68**		
[35]	Romana 2002	Guadalupe	203		2.2	2	317				7	4	4
[43]	El-Gawhary 2017	Egypt	42		0	1	53				0		NE

Abbreviations. Ref.: reference; No.: number; CTR: controls; F/M: female/male; M-TT: methylenetetrahydrofolate reductase TT genotype; SCD: sickle cell disease; VOC: vaso-occlusive crisis; SS: homozygous haemoglobin S; SC/βthal: hemoglobin SC/βthalassaemia; NOS: Newcastle–Ottawa Scale; NE: not evaluable; CBSin68: cystathionine beta synthase in68.

**Table 3:** Demographics and clinical features of the cohort studies on the methylenetetrahydrofolate reductase TT genotype.

Ref.	Author/Year	Ethnicity	SCD		F/M	Age	M-TT + ve	IS + ve	AVN + ve	VOC + ve	NOS
			SS	SC/βthal							
			No.	No.	No.	Years	No.	No.	No.	No.	
*x* ± SD											
[44]	Zimmerman 1998	USA	76	9/1	32/54	23 ± 15	0	16	14		5
[45]	Cumming 1999	Jamaica	96		26/22	7–36	1	48			5
[46]	Driscoll 1999	USA	53		14/39	2–17	1	18			5
[47]	Kutlar 2001	USA	107		66/41	31.9	1		45		4
[48]	Adekile 2001	Kuwait	33	8		12.8 ± 8.6	1		7		6
[49]	Filho 2011	Brazil	94		46/48	6.6	4	24			7
[50]	Jacob 2011 *	Brazil	48	20/23		19–59	2	1		59	4
[51]	Italia 2014 ^@^	India	80	30		20 ± 8	1	6		21	6
[52]	Merghani 2015	Sudan	96		60/36	11 ± 9.2	1			34	5

Abbreviations. Ref.: reference; No.: number; SCD: sickle cell disease; F/M: female/male; M-TT: methylenetetrahydrofolate reductase TT genotype; +ve: positive; IS: ischaemic stroke; AVN: avascular necrosis; VOC: vaso-occlusive crisis; NOS: Newcastle–Ottawa Scale; * 49 patients on hydroxyurea; @: calculations on SS patients only.

**Table 4:** Sensitivity analysis in the adult sickle cell disease/control comparison for plasma homocysteine.

**(A) Sensitivity Analysis by Meta-Regression**				
	**Studies No.**	**CC**	**95% CI**	***p* Value**
Year of publication	12	−0.0006	−0.108, 0109	0.99
Sample size	12	0.101	−0.003, 0.024	0.13
Mean age of SCD participants	12	0.014	−0.124, 0.163	0.80
Female/male ratio	10	0.032	−0.343, 0.279	0.83
NOQAS	12	−0.136	−0.365, 0.092	0.24
**(B) Sensitivity Analysis by Subgroups**				
**Subgroup**	**Studies No.**		**Heterogeneity**	**Effect Size**
	**No.**	**%**	***p* Value**	***p* Value**
**By ethnicity**				
USA	3	87.8	0.0001	0.55
Africa	6	97.8	0.0001	0.74
Other	3	97.5	0.0001	0.83
**by HC assay**				
HPLC	2	99.1	0.0001	0.5
ELISA	5	78.5	0.001	0.0001
Spectrometry	2	81.9	0.01	0.0001
FPIA	2	61.1	0.19	0.005
**by vitamin B12**				
Normal	4	91.0	0.0001	0.47
Low	2	98.6	0.0001	0.20
Not reported	6	93.9	0.0001	0.002
**by folate**				
Normal	5	89.1	0.0001	0.79
Low	1			
Not reported	6	93.9	0.0001	0.002
**by vaso-occlusive crisis**				
Yes	6	98.3	0.0001	0.81
No	6	92.8	0.0001	0.29

Abbreviations. CC: correlation coefficient; CI: confidence interval; SCD: sickle cell disease; NOQAS: Newcastle–Ottawa Quality Assessment Scale; USA: United States of America; HC: homocysteine; HPLC: high-performance liquid chromatography; ELISA: enzyme-linked immunosorbent assay; FPIA: fluorescent polarisation immunoassay.

**Table 5:** Sensitivity analysis in the paediatric sickle cell disease/control comparison for plasma homocysteine.

**(A) Sensitivity Analysis by Meta-Regression**				
	**Studies No.**	**CC**	**95% CI**	***p* Value**
Year of publication	10	0.185	0.133, 0231	<0.0001
Sample size	10	−0.022	−0.041, 0.024	0.027
Mean age of SCD participants	10	−0.286	−0.566, −0.006	0.045
Female/male ratio	9	−1.939	−3.671, −0.206	0.028
NOQAS	12	−0.136	−0.365, 0.092	0.24
**(B) Sensitivity Analysis by Subgroups**				
**Subgroup**	**Studies No.**		**Heterogeneity**	**Effect Size**
	**No.**	**%**	***p* Value**	***p* Value**
**By HC Assay**				
HPLC	7	94.3	0.0001	0.01
ELISA	2	15.3	0.27	0.0001
FPIA	1	na	na	na

Abbreviations. CC: correlation coefficient; CI: confidence interval; SCD: sickle cell disease; NOQAS: Newcastle–Ottawa Quality Assessment Scale; USA: United States of America; HC: homocysteine; HPLC: high-performance liquid chromatography; ELISA: enzyme-linked immunosorbent assay; FPIA: fluorescent polarisation immunoassay.

## Data Availability

Upon request the authors can provide the excel sheets used to collect the data.

## Supplementary Materials

The following supporting information can be downloaded at: https://www.mdpi.com/article/10.3390/ijms232314641/s1, Figure S1: Funnel Plot of Standard Error by Std diff. in means; Figure S2: Plasma homocysteine in sickle cell disease; Figure S3A: Plasma homocysteine by vaso-occlusive crisis; Figure S3B: Plasma homocysteine by vaso-occlusive crisis; Figure S3C: Plasma homocysteine by vaso-occlusive crisis; Figure S4: Methylenetetrahydrofolate reductase 1298CC in sickle cell disease.
