# Supplementary material for: Relevance of Plasma Homocysteine and Methylenetetrahydrofolate Reductase 677TT Genotype in Sickle Cell Disease: A Systematic Review and Meta-Analysis
Source: Int J Mol Sci. 2022 Nov 24;23(23):14641. doi: 10.3390/ijms232314641 (PMC9736045; doi:10.3390/ijms232314641)
Supplement: Supplementary file 1 [file ijms-23-14641-s001.zip › ijms-1960368_v4_Suppl_Fig_proof_2_Verified -1.pdf]

Relevance of Plasma Homocysteine and Methylenetetrahydrofolate Reductase 677TT Genotype in Sickle Cell Disease: A Systematic Review and Meta-Analysis

Paul R. J. Ames, Alessia Arcaro, Matilde Caruso, Maria Graf, Vincenzo Marottoli and Fabrizio Gentile

Supplementary Material

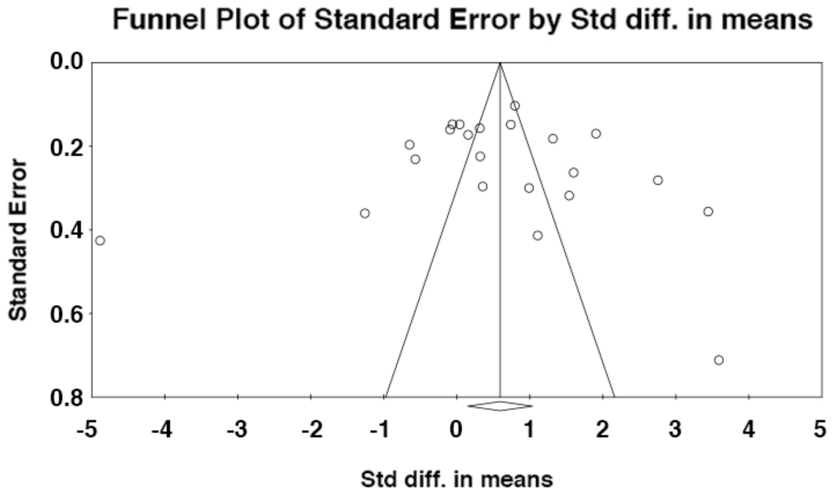

Figure S1. Funnel plot showing a non-significant asymmetry according to the Begg and Mazumdar Rank Correlation Test (Kendall's tau 0.07792,1-tailed *p*-value of 0.30588) and by Egger's Test of the Intercept (Intercept 0.63946, 95% confidence interval (-5.48829, 6.76720), with t=0.21768; 1-tailed *p*-value 0.41494)

Plasma homocysteine in sickle cell disease

Study name	Statistics for each study					
	Standard error	Variance	Lower limit	Upper limit	Z-Value	p-Value
van der Dijs 1998	0.297	0.088	−0.222	0.944	1.215	0.224
Balasa 1999	0.174	0.030	−0.178	0.502	0.934	0.350
R.-Cortes 1999	0.148	0.022	−0.345	0.237	−0.363	0.717
Lowenthal 2000	0.301	0.091	0.406	1.586	3.310	0.001
Balasa 2002	0.149	0.022	−0.245	0.337	0.310	0.756
VanderJagt 2002	0.161	0.026	−0.403	0.229	−0.538	0.591
Dahr 2004	0.158	0.025	0.012	0.630	2.035	0.042
Segal 2004	0.415	0.172	0.301	1.927	2.687	0.007
Abbas 2011	0.319	0.102	0.920	2.171	4.843	0.000
Ajayi 2012	0.232	0.054	−1.017	−0.108	−2.424	0.015
Pandey 2012	0.282	0.080	2.208	3.315	9.776	0.000
Al-Nuzaily 2014	0.713	0.508	2.197	4.993	5.042	0.000
Olaniyi 2014	0.427	0.182	−5.716	−4.042	−11.429	0.000
Nnodim 2015	0.171	0.029	1.577	2.246	11.200	0.000
Abdelsalam 2016	0.104	0.011	0.599	1.006	7.721	0.000
K.-Macaulay 2018	0.197	0.039	−1.028	−0.254	−3.245	0.001
Raouf 2018	0.357	0.128	2.748	4.149	9.652	0.000
Ali 2019	0.225	0.051	−0.116	0.768	1.446	0.148
Higuera 2019	0.362	0.131	−1.960	−0.543	−3.462	0.001
Meher 2019	0.183	0.033	0.964	1.681	7.227	0.000
Uche 2019	0.149	0.022	0.454	1.039	5.000	0.000
Ayoola 2020	0.264	0.070	1.089	2.124	6.081	0.000
	0.227	0.051	0.148	1.037	2.612	0.009

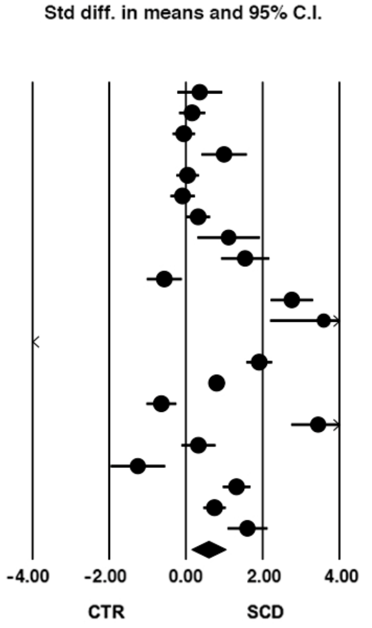

Overall I square 96.2%

Figure S2. Effect size of studies comparing plasma homocysteine in control (CTR) and sickle cell disease (SCD).

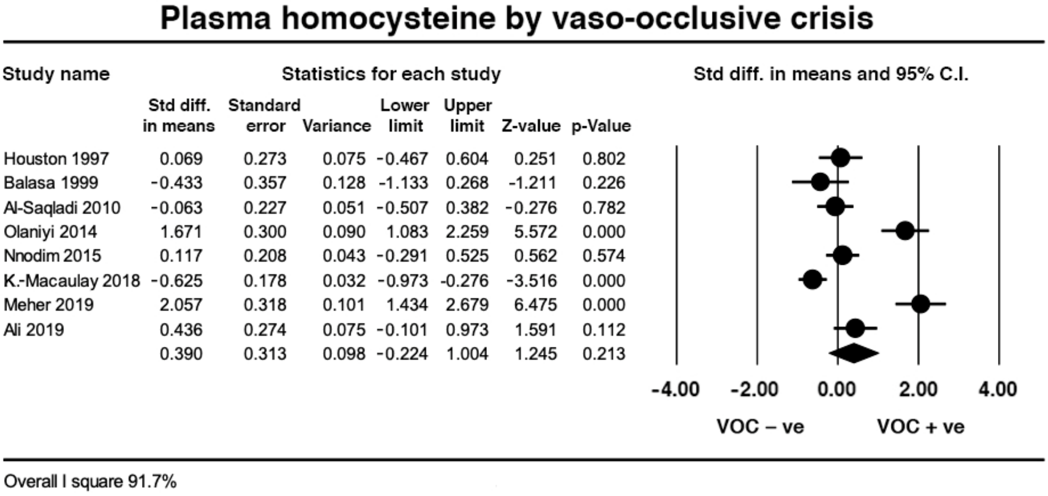

Study name	Std diff. in means	Standard error	Variance	Lower limit	Upper limit	Z-value	p-Value
Houston 1997	0.069	0.273	0.075	-0.467	0.604	0.251	0.802
Balasa 1999	-0.433	0.357	0.128	-1.133	0.268	-1.211	0.226
Al-Saqladi 2010	-0.063	0.227	0.051	-0.507	0.382	-0.276	0.782
Olaniyi 2014	1.671	0.300	0.090	1.083	2.259	5.572	0.000
Nnodim 2015	0.117	0.208	0.043	-0.291	0.525	0.562	0.574
K.-Macaulay 2018	-0.625	0.178	0.032	-0.973	-0.276	-3.516	0.000
Meher 2019	2.057	0.318	0.101	1.434	2.679	6.475	0.000
Ali 2019	0.436	0.274	0.075	-0.101	0.973	1.591	0.112
	0.390	0.313	0.098	-0.224	1.004	1.245	0.213

Figure S3A. Effect size of studies comparing plasma homocysteine in patients in and out vaso-occlusive crisis (VOC).

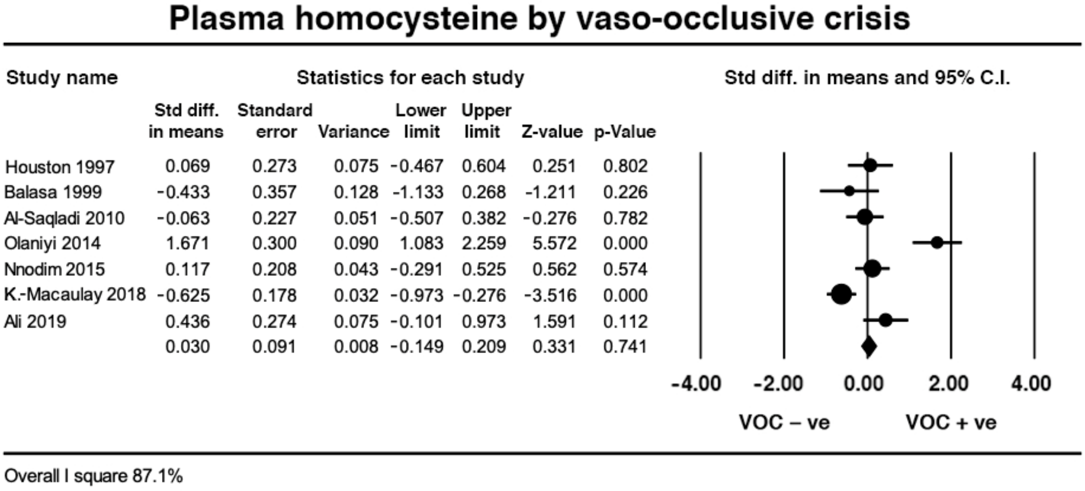

Study name	Std diff. in means	Standard error	Variance	Lower limit	Upper limit	Z-value	p-Value
Houston 1997	0.069	0.273	0.075	-0.467	0.604	0.251	0.802
Balasa 1999	-0.433	0.357	0.128	-1.133	0.268	-1.211	0.226
Al-Saqladi 2010	-0.063	0.227	0.051	-0.507	0.382	-0.276	0.782
Olaniyi 2014	1.671	0.300	0.090	1.083	2.259	5.572	0.000
Nnodim 2015	0.117	0.208	0.043	-0.291	0.525	0.562	0.574
K.-Macaulay 2018	-0.625	0.178	0.032	-0.973	-0.276	-3.516	0.000
Ali 2019	0.436	0.274	0.075	-0.101	0.973	1.591	0.112
	0.030	0.091	0.008	-0.149	0.209	0.331	0.741

Figure S3B. Effect size of studies comparing plasma homocysteine in patients out and in vaso-occlusive crisis (VOC) excluding one study from India (Meher 2019).

Plasma homocysteine by vaso-occlusive crisis

Study name	Std diff. in means	Standard error	Variance	Lower limit	Upper limit	Z-value	p-Value
Houston 1997	0.069	0.273	0.075	-0.467	0.604	0.251	0.802
Balasa 1999	-0.433	0.357	0.128	-1.133	0.268	-1.211	0.226
Al-Saqladi 2010	-0.063	0.227	0.051	-0.507	0.382	-0.276	0.782
K.-Macaulay 2018	-0.625	0.178	0.032	-0.973	-0.276	-3.516	0.000
	-0.324	0.118	0.014	-0.555	-0.094	-2.759	0.006

Std diff. in means and 95% C.I.

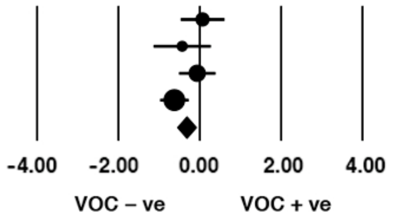

Overall I square 52.7%

Figure S3C. Effect size of studies comparing plasma homocysteine in patients out and in vaso-occlusive crisis (VOC) excluding one study from India (Meher 2019) and three studies from Nigeria (Olaniyi 2014, Nnodim 2015, Ali 2019)

Methylenetetrahydrofolate reductase 1298CC in sickle cell disease

Study name	Odds ratio	Lower limit	Upper limit	Z-Value	p-Value
Al-Absi 2006	2.791	1.300	5.991	2.633	0.008
Nefissi 2018	5.939	0.522	67.644	1.435	0.151
Adelekan 2019	0.495	0.044	5.549	-0.571	0.568
	2.466	1.010	6.017	1.983	0.047

Odds ratio and 95% C.I.

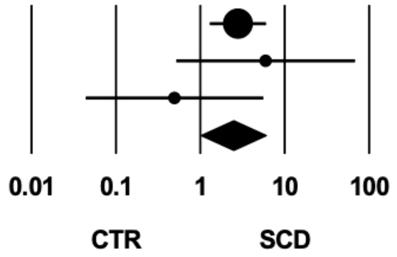

Overall I square 12.4%

Figure S4. Effect size of the pooled prevalence of the methylenetetrahydrofolate reductase 1298CC in controls (CTR) and in sickle cell disease (SCD).
